# Exploring the Relationship of Cognitive Disengagement Syndrome and Attention Deficit/Hyperactivity Disorder with Emotional Dysregulation: A Twin Study in Childhood and Adolescence

**DOI:** 10.3390/mps8040094

**Published:** 2025-08-11

**Authors:** Simona Scaini, Stefano De Francesco, Ludovica Giani, Marco Battaglia, Emanuela Medda, Corrado Fagnani

**Affiliations:** 1 Child and Youth Lab, Sigmund Freud University, 20143 Milan, Italy; defrancesco.phd@milano-sfu.it (S.D.F.); l.giani@milano-sfu.it (L.G.); 2 Italian Psychotherapy Clinics, Child and Adolescent Unit, Studi Cognitivi Group, Corso San Gottardo 5, 20121 Milan, Italy; 3 Centre for Addiction and Mental Health, Toronto, ON M6J 1H4, Canada; marco.battaglia@utoronto.ca; 4 Department of Psychiatry, University of Toronto, Toronto, ON M1C 1A4, Canada; 5 Cundill Centre for Child and Youth Depression, Toronto, ON M5T 1R8, Canada; 6 Center for Behavioural Sciences and Mental Health, Italian Twin Registry, Istituto Superiore di Sanità, 00161 Rome, Italy; emanuela.medda@iss.it (E.M.); corrado.fagnani@iss.it (C.F.)

**Keywords:** cognitive disengagement syndrome, sluggish cognitive tempo, ADHD, emotion regulation, twin study

## Abstract

Data on the genetic and environmental factors underlying the co-occurrence of Cognitive Disengagement Syndrome (CDS), Attention Deficit Hyperactivity Disorder (ADHD), and Emotional Dysregulation (ED) are limited. This study aimed to explore the nature of the associations between CDS, ADHD with ED, and to assess the role of shared etiological factors in explaining their comorbidity. We analyzed a sample of 400 Italian twin pairs aged 8–18, from Northern Italy and enrolled in the Italian Twin Registry. Bivariate genetic analyses were conducted using parent-rated CBCL scores for CDS, ADHD, and ED. For both CDS–ED and ADHD–ED associations, the best-fitting models were Cholesky AE models (−2LL = −849.167 and −339.030, respectively; *p* > 0.05), suggesting that the covariation was mainly due to additive genetic factors (CDS–ED—A = 0.81, 95% CI [0.66–0.95]; ADHD–ED—A = 0.86, 95% CI [0.75–0.95]). More than half of the genes were shown to be shared among the phenotypes. Non-shared environmental contributions were smaller (CDS–ED—E = 0.19, 95% CI [0.05–0.34]; ADHD–ED—E = 0.14, 95% CI [0.05–0.25]), indicating interrelated but distinct constructs. Despite some limitations, particularly the exclusive use of the CBCL, findings highlight the importance of monitoring ED symptoms in individuals with CDS or ADHD, and vice versa.

## 1. Introduction

Cognitive Disengagement Syndrome (CDS), previously known as “Sluggish Cognitive Tempo”, refers to a distinct and persistent collection of symptoms that are developmentally inappropriate. CDS can be characterized by at least two dimensions [1,2,3]. The first dimension involves cognitive symptoms, where attention and mental processing appear disengaged or decoupled from the ongoing external context. This is evident through traits such as staring, daydreaming, mental “fogginess”, withdrawal, and a sleepy appearance. The second dimension includes motoric manifestations and hypoactivity, represented by underactivity, periods of passive or sedentary movement, and slow, reduced, or delayed motor movements [1,2,3].

Although initially arising from studies focused on the dimensionality of Attention Deficit and Hyperactivity Disorder (ADHD), it is now clear that CDS symptoms are distinct but closely linked to the inattentive symptoms of ADHD [1,4]. Consistently, relatively recent research has highlighted the distinction between CDS and ADHD, indicating that CDS symptoms are discernible from both the inattentive (ADHD-INP) and hyperactive–impulsive (ADHD-HIP) subcategories of ADHD [5,6,7,8]. Several features support a distinction between CDS and the classical ADHD profile.

The inattention in CDS seems to be mainly driven by distraction towards internal stimuli, leading to increased “daydreaming” and resembling the rumination that is proper of Internalizing Disorders (INT). In contrast, ADHD’s inattention is more influenced by external stimuli [9,10]. Longitudinally, CDS symptoms show lower stability than ADHD symptoms, and CDS predicts lower adherence to, and the limited effectiveness of, methylphenidate compared to ADHD [11].

People with ADHD vs. CDS also exhibit different well-being profiles, suggesting that CDS involves a distinct set of challenges in terms of emotional well-being, social engagement, and behavioral tendencies.

CDS symptoms emerge in early childhood [12] and predict relatively specific functional outcomes, including poor future academic functioning and social difficulties [1,4,9,13,14]. However, CDS and ADHD often co-occur, particularly when the inattentive component is prominent, and with symptoms characteristic of the INT features of ADHD [1,15].

Twin studies have been pivotal in elucidating the etiopathogenic factors of CDS, distinguishing it from ADHD, and providing evidence for its strong association with INT symptoms.

The classical twin design involves comparing monozygotic (MZ) twins, who originate from a single fertilized egg and are therefore genetically identical, with dizygotic (DZ) twins, who result from two separate fertilizations and share, on average, 50% of their segregating genes—similar to typical siblings [16]. In this framework, estimates of genetic and environmental influences on the phenotypes under investigation are derived from intrapair correlation coefficients. If the correlation for MZ twins (rMZ) closely resembles that for DZ twins (rDZ), genetic contributions to the trait in question are likely minimal. Conversely, a substantially higher rMZ compared to rDZ suggests a genetic influence on the phenotype [16,17]. To further disentangle these influences, Structural Equation Models (SEM) are employed, allowing researchers to estimate the relative contributions of genetic and environmental factors to both the variance of individual traits and covariance between traits [16].

Among twin studies, the work by Moruzzi et al. [8] is the one that has most significantly contributed to providing substantial support that ADHD and CDS are distinct—albeit highly correlated—phenomena. Specifically, they were the first to demonstrate that, while CDS, ADHD-INP, and ADHD-HIP were genetically and environmentally correlated, they remained etiologically distinct, with CDS exhibiting the lowest heritability. A subsequent twin study [18] confirmed this pattern, identifying similar latent factors underlying both phenotypes, though reporting slightly higher heritability for CDS, which was still lower than for ADHD.

Regarding the relationship with INT symptoms, CDS is associated with increased anxious and depressive symptoms, social difficulties, group isolation, and a preference for loneliness [19]. The only twin study on this topic, conducted by our group [20], found significant associations between CDS and somatic/generalized anxiety. A Common Pathway model best explained these associations, indicating a shared latent vulnerability influenced by both genetic and environmental factors. This supports the idea that CDS and anxiety, while clinically distinct, may stem from overlapping etiological mechanisms [20].

Evidence on the relationship between CDS and externalizing (EXT) symptoms is less consistent. Studies reported positive, negative, or no associations between CDS and EXT behavior when controlling for ADHD-INP [21,22,23]. Moreover, existing associations between CDS and EXT become non-significant, or even negative, when ADHD symptoms are injected as a covariate [1].

One of the conditions pertaining to the EXT domain most frequently associated with ADHD in the literature is emotional dysregulation (ED). Emotional regulation processes can commonly be defined as a series of processes concerning physiological and behavioral regulation in response to an emotional stimulus [11,24]. The co-occurrence rates between ADHD and emotional regulation deficits are estimated to be around 38% generally in non-clinical populations, and can range from 24% to 50% in clinical populations. Several hypotheses have been proposed to explain the association between these two conditions. A leading one suggests that it stems from a deficit in executive inhibition, with recent findings pointing to a working memory impairment that may influence inhibitory mechanisms [24].

Emotional regulation deficits in children and adolescents with ADHD increase the risk of developing comorbid INT and EXT disorders, and this association appears to persist across the lifespan. Emotional symptoms tend to shift from overt manifestations in childhood, such as difficulties in following rules and managing peer relationships, to more complex presentations in adolescence and adulthood, where they are often linked to anxiety and depression [25,26].

As for CDS, it has also been consistently associated with lower self-reported emotional regulation. Specifically, a connection between CDS and poor emotional regulation has been found in children and adults [6,9,11], even after controlling for ADHD-INP, ADHD-HIP and INT symptoms [27,28]. This association appears to be primarily explained by the difficulties related to mental fogginess, which may impair the processing of both external stimuli and internal emotional states. This, in turn, may trigger a cycle of emotional dysregulation, particularly affecting the expression of emotions, which becomes dysfunctional [6].

Although ED is more strongly associated with ADHD than with CDS, recent studies suggest that it plays a mediating role between these two conditions and INT symptoms. This finding sheds light not only on the nosology of ADHD but also on the importance of emotional regulation in the deficits typically observed in CDS.

The association of ADHD with CDS and ED led Sonuga-Barke et al. [29], in a recent review on the topic, to conclude that these may even constitute a diagnostic subtype of ADHD. In the conclusions of their work, they call for further studies to clarify the genetic and environmental factors responsible for this relationship, aiming to better understand ADHD in its heterogeneous manifestations.

Very few twin studies have so far aimed to shed light on the etiological factors underlying this association. Regarding the ADHD–ED association, the only twin study conducted on a general population sample to date is that by Merwood et al. [30]. In this study, the authors found that more than half of the shared variance between ADHD and emotional regulation problems was attributable to shared genetic influences, linked to a unique, highly heritable latent susceptibility factor [30]. It is worth noting, however, that the authors in this case used an extremely narrow measure consisting of only four items from the Conners 10 [31] to assess ED, which did not account for the heterogeneity of the behavioral manifestations associated with this phenotype.

Regarding the CDS and ED, to our knowledge, research has not yet investigated the etiology of their covariation [32].

Given these premises, in the present study, we aimed to address existing literature gaps by examining a twin sample of children drawn from the general population. Our primary objective was twofold: firstly, to evaluate the association of ADHD and CDS with ED, and secondly, to explore the potential role of shared etiological factors in the observed comorbidity and risk overlap among these phenotypes, using the twin study design.

Considering the nature of ED and its manifestation across various psychopathological dimensions, a more comprehensive understanding of the genetic and environmental contributions to its expression and co-occurrence with ADHD and CDS could significantly enrich the existing literature. Moreover, such insights have the potential to foster the development of psychological interventions tailored to address ED problems in children/adolescents with ADHD and/or CDS symptoms. Our study seeks to contribute information that can advance the field and enhance the prospects for effective therapeutic strategies.

## 2. Materials and Methods

### 2.1. Participants

This study is a part of a project involving the population-based Italian Twin Registry (ITR). The procedures that resulted in the establishment of the ITR are detailed elsewhere [33]. Currently, the Registry contains the detailed information of approximately 30,000 twins from various regions of Italy. It is actively exploited for national and international research projects, with a specific emphasis on behavioral and psychiatric genetics [34].

In the present study, only twins already enrolled in the ITR, with available information on the targeted phenotypes and within the targeted age range, were included. Specifically, this study originates in a recruitment that took place in 2003, involving 2015 families with potential twins born between 1986 and 1995 and residing in the provinces of Milan and Lecco (Northen Italy). These families were invited to participate in a survey. Of them, 973 families (48%) confirmed the actual presence of a twin pair among their children. Among these, 707 agreed to take part in various types of surveys, and 407 families (57.5%) participated in a psychometric study that included questionnaires to be completed by the children and one parent [35]. Seven twin pairs were excluded from the present study due to missing data, leaving 400 pairs included in the analysis. The participants were between 8 and 18 years old (mean age 14 ± 2.6), with a homogeneous distribution of sex assigned at birth [47% assigned male at birth (AMAB), 53% assigned female at birth (AFAB)]. As an inclusion criterion, at the time of recruitment, none of the participants carried certified mental/physical handicaps requiring special attention, such as differential academic programs or a remedial teacher. The determination of the twins’ zygosity was established using the Goldsmith parent-rated questionnaire. The instrument comprises 29 items designed to assess physical, medical, and behavioral similarities between twins, with the aim of estimating whether they are monozygotic (MZ) or dizygotic (DZ). Based on participants’ responses, a validated algorithm is applied, which relies on both the quantitative evaluation of the reported parameters and qualitative assessments, such as the detection of inconsistencies [36]. This method has been shown to be useful in determining zygosity, with an estimated accuracy of approximately 94%. Using this procedure, 144 MZ and 266 DZ twin pairs were identified within the study sample.

### 2.2. Behavioral Measures

Information on the phenotypes under investigation was obtained through the Child Behavior Checklist questionnaire (CBCL/6-18; [37]). CBCL/6-18 is a parent-report questionnaire that is widely used to identify emotional and behavioral problems in children aged 6 to 18 years old. It consists of 113 items, scored on a three-point Likert scale (0 = absent, 1 = occurs sometimes, 2 = occurs often).

Depending on how the items are summed together, two types of scales can be obtained—syndromic scales, which have 8 levels and were originally empirically built through exploratory and confirmatory factor analyses; DSM-Oriented scales, which have 6 levels and are obtained by grouping items in such a way that the resulting symptoms in each subscale are consistent with those reported in the DSM-IV [38].

Within the present study, the scores related to ADHD symptoms were obtained through the DSM-Oriented scale “Attention deficit/Hyperactivity problems”, which includes 7 items. Regarding CDS, there is currently no instrument exclusively designed for the measurement of this syndrome. For this reason, in this study, information regarding CDS levels in the sample was obtained through a specific scale of the CBCL, which includes 4 items reflecting the characteristics of this condition (items 13 “is confused or seems to have their head in the clouds,” 17 “daydreams, gets lost in their thoughts,” 80 “stares into space,” and 102 “is not very active, slow in movements, not energetic”) [39]. Data regarding participants’ levels of emotional dysregulation were obtained through the CBCL-DP (Dysregulation Profile). The CBCL-DP has been considered as a potential diagnostic tool for identifying children exhibiting specific features [40]. It derives from the sum of the syndromic scales “Attention” (10 items), “Aggression” (18 items), and “Anxious/Depressed” (13 items) [41]. The CBCL-DP has been identified as a reliable indicator of concurrent conditions such as ADHD, Oppositional Defiant Disorder (ODD), and Mood Disorder (MD) [42]. In addition, it has been proven to be a good indicator of a worse prognosis when in the presence of an ADHD diagnosis, as demonstrated by longitudinal studies. In particular, in a comprehensive investigation involving children diagnosed with ADHD and tracked into late adolescence, the findings revealed that a CBCL-DP score exceeding 180 during the initial assessment predicted compromised psychosocial functioning, an increased likelihood of psychiatric hospitalization, and a subsequent diagnoses of conduct disorder, depression, and bipolar disorder at follow-up [41]. 

ADHD and ED subscales showed good reliability in our sample (Cronbach’s alpha = 0.77 for ADHD and 0.89 for ED); a lower Cronbach’s alpha was found for the CDS subscale (0.50), as also reported in a previous study on basically the same sample and using the same measurement tool [8].

### 2.3. Statistical Analyses

#### 2.3.1. Preliminary Analyses

First, descriptive statistics were calculated for each scale, and independent samples *t*-tests were performed to identify potential differences in mean scores based on sex and zygosity. Before proceeding with model-fitting analyses, all scales were log-transformed to meet the normality criterion required for the maximum-likelihood estimation method. The scores of each scale were regressed on participants’ age and sex, and residuals were used in subsequent model-fitting analyses, which were carried out in the OpenMx software (ver. 2.21.13) [43].

#### 2.3.2. Model Fitting Analyses

In the present study, the CBCL was the only instrument used to estimate the phenotypes under investigation. This introduces an issue related to item overlap among the subscales. Specifically, the CBCL-DP “Attention” subscale includes 5 items shared with the Attention Deficit/Hyperactivity Problems scale (i.e., items 4, 8, 10, 41, 78) and 3 items shared with the CDS subscale (i.e., items 13, 17, 80).

To address this issue and avoid inflating correlations among the constructs examined, model-fitting analyses were implemented using two separate bivariate models. The first included Attention Deficit/Hyperactivity Problems and CBCL-DP subscales, excluding the shared items; the second incorporated CDS and CBCL-DP subscales, again excluding the shared items.

First, through the application of two saturated models, phenotypic or within-twin/cross-trait correlations (i.e., between different subscales within a twin individual), cross-twin/within trait correlations (i.e., between twin and cotwin for the same subscale, stratified by zygosity) and cross-twin/cross-trait correlations (i.e., between a given subscale in a twin and a different subscale in the cotwin, stratified by zygosity) were estimated [17].

Specifically, at first, two free saturated models were applied, requiring a number of free parameters equal to the number of observed statistics. Subsequently, the following constraints were applied to both models: (i) equal means and variances for each phenotype for twin1 and twin2, MZ and DZ, based on the assumption that twins, as individuals, are representative of the general population; (ii) the same cross-twin/cross-trait covariances regardless of twin order (i.e., covariance between trait-x in twin1 and trait-y in twin2 equal to covariance of trait-y in twin1 and trait-x in twin2) within each zygosity group, assuming that the relation between twins of the same pair is symmetrical [44].

Afterward, bivariate biometric models were applied to further investigate the genetic and environmental contributions to the covariance between traits. Bivariate twin designs allow for the partitioning of the variance and covariance of traits into distinct components—additive genetic influences (A), which encompass the cumulative effects of individual genetic variants acting independently; non-additive genetic influences (D), referring to interactions either between alleles at the same locus (dominance) or across different loci (epistasis); shared environmental influences (C), which include environmental factors experienced jointly by both twins—such as familial upbringing, socioeconomic context, parental practices, or prenatal conditions like hormonal exposures; and non-shared environmental influences (E), representing environmental factors that are unique to each twin—such as peer interactions, personal habits, or infections—as well as measurement error, which captures variance not attributable to identifiable sources [17].

Where significant correlations between behavioral measures are observed, a Cholesky model can be applied to the data to assess the contribution of shared genetic and environmental factors to the observed correlations. For *n* variables, a Cholesky decomposition includes *n* independent genetic and environmental factors; the first factor loads on all traits, the second one loads on all traits but the first, the third factor loads on all traits except the first two, and so on [20].

For both bivariate models, the analysis process began by applying a full model to the data, and then progressively eliminating genetic and environmental components that contributed negligibly or non-significantly to the variance and covariance of the examined traits. Thus, by adhering to the principle of parsimony and progressively reducing the number of explanatory variables, the best-fitting model was selected. Following this principle, models that display fewer latent variables are favored over more comprehensive ones, provided they do not result in a significant worsening of fit. This selection process involves choosing models with the lowest Akaike Information Criterion (AIC), the lowest Bayesian Information Criterion (BIC) and, concurrently, a not significant χ^2^ test [45]. In our case, following the recommendations of Baker et al. [46] and Kendler et al. [47] suggesting that the BIC provides better accuracy than the AIC in identifying the best-fitting model in the case of particularly complex models, when two models showed very similar AIC values, the one with the lower BIC was chosen.

## 3. Results

Table 1 reports the mean values of the ADHD, CDS and ED scales in twins considered as individuals by sex and zygosity. Comparisons by *t*-tests yielded higher scores in ADHD and ED features for boys compared to girls, while no such difference was found for CDS scores. Moreover, as expected, no difference in mean values was found in relation to zygosity for any of the traits considered.

Table 2 presents the estimated correlations from the bivariate saturated models. All phenotypes were highly correlated with each other, particularly ADHD and ED, as is consistent with the existing literature. For both the models tested, cross-twin/within-trait correlations for ADHD and CDS were more than twice as high in MZ compared to DZ pairs, while for ED, the same correlations were still higher in MZ pairs but to a lesser extent compared to the other two phenotypes. The same pattern was observed for the cross-twin/cross-trait correlations, which were systematically higher in MZ pairs, suggesting genetic overlap between the phenotypes.

These results suggest that ADHD and CDS may be influenced by non-additive genetic factors, whereas shared environmental effects might play a role in ED.

Following these considerations, two bivariate models were later applied to the data during the biometric analysis phase. These models accounted for both non-additive genetic effects and shared environmental influences.

### 3.1. ADHD–ED Model

**Figure 1:**
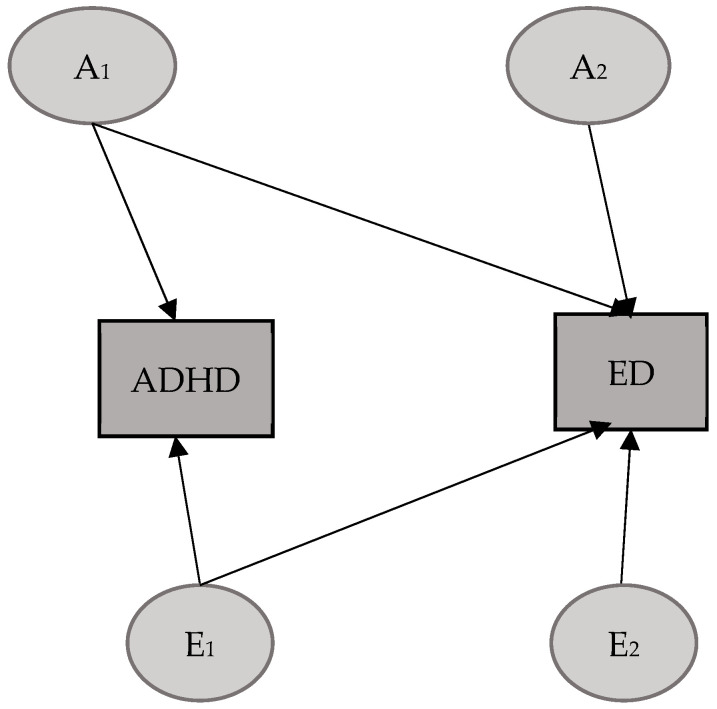
Best-fitting model ADHD–ED. A= additive genetic factors; E = unique environmental factors.

For the model in which the ADHD and CBCL-DP subscales were considered (after excluding overlapping items), the correlation pattern led us to select a Cholesky mixed model, accounting both for non-additive genetic effects (D) for ADHD and the influence of the shared environment (C) for ED. As shown in Table 3, we first tested the mixed model in its full form. After observing that the contribution of D was not significant (95% CIs: [0, 0.21] for d11), we tested Model 2, which excluded this component. Model 2 revealed that the contribution of C to ED was also non-significant (95% CIs: [0, 0.19] for c33), which led us to test a more parsimonious AE model (Model 3). This model (Figure 1) turned out to be the best-fitting one.

As shown in Table 4, the results for ADHD variance indicate an almost equivalent contribution from genetics and unique environmental factors, with the latter accounting for a slightly larger portion of the total. Regarding ED, the results show a predominant contribution of additive genetic factors to the total variance (76%), with a significantly lower contribution from the unique environment (24%). The same applies to the covariance between ADHD and ED, where additive genetic factors again seem to play a key role, explaining as much as 86% of the covariance. In this case, the correlation coefficients also indicate an almost complete overlap of the shared genetic factors between the two traits (92%), and a minimal overlap of the unique environmental factors.

### 3.2. CDS–ED Model

As in the previous case, for the model including CDS and the ED variable obtained by removing overlapping items from the CBCL-DP, it was decided to initially select a mixed Cholesky model, including D for CDS and C for ED. Just as in the previous model, in this case, after observing that the contribution of D was not significant (95% CIs: [0, 0.25] for d11), we excluded it and tested Model 2, as shown in Table 5. Once it was determined that, once again, the contribution of C for ED was also not significant (95% CIs: [0, 0.30] for c33), we proceeded to test an AE model (Model 3). The results of the model fitting analysis suggest that Model 3 offered a better fit to the data, albeit much less conclusively than in the previous case, as the maximum likelihood test in this instance approached the threshold of statistical significance (*p* = 0.07).

**Figure 2:**
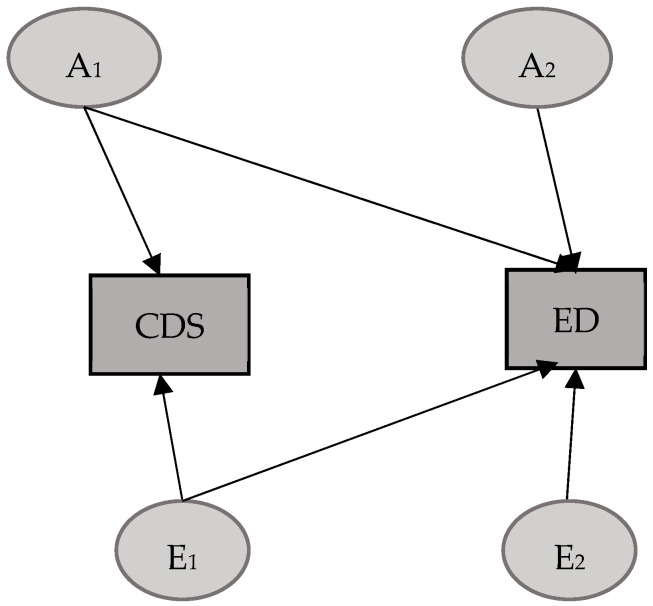
Best-fitting model CDS–ED. A = additive genetic factors; E = unique environmental factors.

The genetic and environmental standardized components obtained from the best-fitting model (Figure 2) are reported in Table 6.

As shown, the genetic and environmental contributions to the variance of CDS appear to be primarily attributable to unique environmental factors. The opposite pattern, however, is seen for ED, the variance estimates of which align with those of the first model.

In this case as well, the covariance between phenotypes appears to be almost entirely determined by additive genetic factors (81%), with a minimal contribution from the unique environment. Furthermore, the genetic correlation coefficient indicates a large overlap of shared genetic factors (81%), albeit to a lesser extent than that observed between ADHD and ED. Meanwhile, the overlap of unique environmental factors between the two phenotypes seems to be the same as in the previous model.

## 4. Discussion

The primary aim of this study was to examine the relationships between CDS, ADHD, and ED, assessing the role of shared etiological factors in explaining their comorbidity. In both cases, data showed positive, moderate to high phenotypic correlations that support constructs’ psychometric separation together with a degree of reciprocal interdependence. The strongest association was found between ED and ADHD, which is consistent with findings of several previous studies [1,4,9], but the degree of association between ED and CDS was also notably high.

The results of the model-fitting analysis indicate that, in both cases, the phenotypic associations can be mostly explained by genetic factors, with a small contribution of the unique environment. Focusing on the two models individually, in the ADHD–ED model, our heritability estimate of ADHD is somewhat lower than that generally shown in the literature. However, it is in line with the results of a previous study conducted by our group on basically the same sample, focusing on the CBCL/6-18 DSM-Oriented Scales [38].

These findings confirm those reported by Merwood et al. [30] and expand on them, as we used a measure that more accurately captured the various emotional and behavioral manifestations of ED. In their study, the authors conducted a multivariate analysis including ADHD-HIP, ADHD-INP, and emotional dysregulation problems as phenotypes. They found that the best-fitting model involved a single latent susceptibility factor, with high heritability and a smaller contribution from unique environmental factors, alongside the concurrent presence of both genetic and unique environmental factors specific to each phenotype, suggesting an incomplete overlap between the individual dimensions.

Similarly, in our case, the results show that the covariance between the two phenotypes is determined at 86% by additive genetic factors, with genetic correlations indicating that a consistent amount of genes are shared between the two phenotypes. This is in line with the existing literature suggesting that ED may indeed reflect a component of ADHD, while still remaining a distinct symptomatic dimension [29].

Concerning the CDS–ED model, the variability in CDS appears to be attributed to both genetic (29%) and environmental factors (71%). These findings align with prior research contributions that have reported CDS heritability ranging between 28% and 66% [8,18,20]. Regarding ED, the estimates of phenotypic variance observed are almost perfectly comparable to those identified in the previous model, and confirm a predominant genetic contribution to the total variance. This coincidence in estimates leads us to believe that the items alternately removed from the CBCL-DP scale in the two models likely did not have a significant impact when estimating the etiological factors underlying ED. With respect to the covariance between CDS and ED, as in the previous case, the best-fitting model showed that most of the covariancve is determined by additive genetic factors (81%), with minimal contributions from unique environmental influences.

In summary, the two tested models appear to support the framework proposed by Sonuga-Barke et al. [29] regarding the ADHD–ED association, and suggest that these findings may also be extended to the CDS–ED relationship. The covariance estimates observed in the models indicate that ED shares a substantial biological vulnerability with both ADHD and CDS, with a pronounced overlap in the genetic factors underlying this shared vulnerability (r_a_ = 0.92 and r_a_ = 0.81, respectively). These findings suggest that ED may function as a transdiagnostic feature of both conditions while remaining a distinct symptomatic dimension. They also extend existing evidence on the association between CDS and ED [11,27,48], by clarifying, for the first time, the etiological factors underlying this link, and offering clinically relevant insights for the treatment of CDS.

In fact, to date, only a limited number of studies have examined the effectiveness of psychotherapeutic interventions in reducing symptoms associated with CDS and its related impairments. Given the identified link between CDS and ED—strikingly similar to the well-documented association between ADHD and ED—it can be hypothesized that, in a similar manner, treatment approaches for both ADHD and CDS may benefit from the integration of techniques aimed at enhancing emotional awareness and regulation. This insight not only reinforces current evidence-based interventions for ADHD, but also contributes to the development of preliminary clinical guidelines for the treatment of CDS, which are currently lacking. Cognitive–behavioral therapy (CBT), recognized as the gold-standard non-pharmacological treatment for ADHD [49], may represent a promising approach for CDS as well. In the context of ADHD, CBT typically includes psychoeducation, behavioral strategies, and parent/teacher training. Our findings suggest that these interventions could be further optimized by placing greater emphasis on emotion regulation strategies—such as relaxation techniques, mindfulness practices, and the cognitive restructuring of automatic negative thoughts. Although these techniques are already included in standard CBT protocols for ADHD [50,51,52], the current results highlight the potential value of further enhancing them in interventions targeting both ADHD and CDS.

Overall, from a clinical perspective, the data suggest that CDS, ADHD, and ED are distinct psychopathological constructs. The association of CDS and ADHD with ED is primarily influenced by biological determinants but also by unique environmental risk factors. Some of these environmental factors may include peer interactions and adverse school experiences, both known to increase the risk of developing psychopathological symptoms [20]. It is well known, for instance, that traumatic experiences can be a potential environmental risk factor associated with emotional dysregulation issues. In fact, children with histories of trauma often exhibit significant self-regulatory problems, which can frequently co-occur with both INT and EXT symptoms [53]. Symptoms associated with traumatic experiences can also negatively affect the attentional abilities of children and adolescents [54]. These findings further suggest that another type of therapeutic approach that may help alleviate the symptoms of ADHD, ED, and CDS could include trauma-focused therapies.

However, future research is warranted to further investigate the effects of specific environmental factors in explaining this covariation. Additionally, molecular studies can contribute to understanding the common genetic etiology by identifying specific genes influencing the liability to all the phenotypes considered.

## 5. Limitations

When interpreting our findings, it is important to acknowledge certain potential limitations. The primary limitation was the use of a single instrument (CBCL) for assessing the phenotypes under examination. This choice was primarily due to the lack of available data from the target population based on alternative psychometric tools. This limitation is particularly relevant in the case of ED, for which validated assessment instruments in Italian do exist, but were not available in our dataset. In the case of CDS, however, the issue is less attributable to our methodological choices, as no validated instruments for assessing CDS in the Italian population are currently available in the literature.

This raises a key concern regarding the reliability of the CBCL subscale used to assess CDS, which might be considered poor [55], as also shown by the low Cronbach’s alpha in our study. Such limited reliability might have impacted the correlation between CDS and ED, ultimately affecting twin analyses.

Alongside this critical aspect, it is important to note that, given that CDS features may be more closely related to INT than EXT symptoms, relying solely on parent-reported measures could lead to underestimates of the true severity of the syndrome. Indeed, it is well known that for INT symptoms, self-report questionnaires are the most reliable tools for their assessment, unlike EXT, which are better detected by external raters [56].

Certainly, the use of multiple raters and instruments would have allowed for a more specific characterization of the traits under investigation and a more accurate estimation of their associations. Future studies should build on our findings by employing more precise and syndrome-specific measures for both ED and CDS. This would help avoid item overlap, which in turn would allow for the application of more refined multivariate models capable of simultaneously examining the genetic and environmental etiological factors underlying ADHD, CDS, and ED, and thus analyzing their associations with greater accuracy.

Another limitation of our study concerns the relatively limited sample size, which may have hindered the detection of a significant contribution from non-additive genetic effects (D). This is particularly relevant given that the identification of such effects often requires larger samples to accurately estimate their impact on phenotypic variance [57]. In our case, this limitation may have affected the ability to detect non-additive influences in the association between ADHD and ED, which have been reported in previous literature [30]. The limited sample size also restricted our ability to explore sex differences in symptom etiology. The relevance of this limitation arises from the recognition that sex significantly influences the developmental trajectories of INT-EXT problems, as well as those of CDS, and that there are notable differences in prevalence rates [58,59].

Moreover, problem behaviors were assessed at only one point in time, which made it difficult to distinguish unique environmental effects from measurement errors [35].

Another consideration is that our estimates were derived from a specific age range only. Prior studies have shown that the heritability of INT-EXT symptoms tends to increase with age [60,61], and that both the severity and prevalence of CDS may differ between childhood and adulthood [62]. These data underscore the importance of considering developmental aspects when interpreting the contributions of genetic and environmental factors. In light of these limitations, future studies should aim to expand on our findings by employing sufficiently large samples to allow for stratified analyses by sex, in order to better capture its potential role in moderating the etiological factors underlying these phenotypes. Regarding age, longitudinal designs are strongly recommended to examine potential developmental changes in the genetic and environmental influences on CDS—particularly to determine whether its etiological patterns follow those already identified for INT-EXT symptoms over time. A longitudinal approach would also help to address limitations related to measurement error by providing more stable estimates across time points. Alternatively, cross-sectional cohort studies with samples sufficiently large to permit age stratification could be used to compare etiological patterns across developmental stages.

## 6. Conclusions

In conclusion, our research found significant correlations of CDS and ADHD with emotional dysregulation. The twin method used in the current study has shed light on the etiological factors underlying these associations, proposing common genetic and environmental factors for both ADHD–ED and CDS–ED. These results may hold substantial value in clinical settings, as they would encourage clinicians to monitor signs of emotional dysregulation when dealing with patients presenting symptoms attributable to CDS or ADHD, and vice versa.

## Figures and Tables

**Table 1:** Demographic characteristics, and CBCL/6–18 ADHD, CDS and ED values, by sex and zygosity.

	ADHD Min–Max: 0–13	CDS Min–Max: 0–8	ED Min–Max: 0–58
	Mean (SD)	Mean (SD)	Mean (SD)
Total sample (N = 800)	2.9 (2.7) *	0.8 (1.1) **	11.7 (8.7) ***
			
Males (N = 376)	3.5 (3.9)	0.8 (1.1)	12.7 (9.2)
Females (N = 424)	2.3 (2.4)	0.8 (1.1)	10.8 (8.1)
	*p* < 0.01	*p* = 0.94	*p* = 0.04
MZ (N = 288)	2.8 (2.6)	0.8 (1.0)	11.7 (8.5)
DZ (N = 512)	3.0 (2.7)	0.8 (1.1)	11.7 (8.7)
	*p* = 0.32	*p* = 0.57	*p* = 0.89

Notes. *p* = *t*-test of means; MZ = monozygotic twins; DZ = dizygotic twins; ADHD = Attention Deficit/Hyperactivity Disorder; CDS = Cognitive Disengagement Syndrome; ED = emotional dysregulation; * ADHD value at the 95th percentile within the sample = 8.0; ** CDS value at the 95th percentile within the sample = 3.0; *** ED value at the 95th percentile within the sample = 28.0.

**Table 2:** Twin correlations among the study phenotypes.

ADHD–ED			CDS–ED		
Within-Twin/Cross-Trait Correlations					
	ADHD	ED		CDS	ED
ADHD	1	-	CDS	1	-
ED	0.62 *	1	ED	0.48 *	
Cross-Twin/Within-Trait correlations					
	ADHD	ED		CDS	ED
MZ	0.51 *	0.74 *	MZ	0.37 *	0.75 *
DZ	0.15 *	0.55 *	DZ	0.10 *	0.54 *
Cross-Twin/Cross-Trait correlations					
MZ	ADHD	ED	MZ	CDS	ED
ADHD	1	-	CDS	1	-
ED	0.51 *	-	ED	0.37 *	1
DZ	ADHD	ED	DZ	CDS	ED
ADHD	1	-	CDS	1	-
ED	0.37 *	1	ED	0.29 *	1

ADHD = Attention Deficit/Hyperactivity Disorder; ED = emotional dysregulation; CDS = Cognitive Disengagement Syndrome; MZ = monozygotic; DZ = dizygotic; *: *p* < 0.05.

**Table 3:** Bivariate model-fitting analysis with sub-model comparisons for ADHD and ED.

Model	ctm	ep	−2LL	df	AIC	BIC	diffLL	Δdf	*p*
Model ACDE	-	14	−342.594	1484	−314.594	−258.714	-	-	-
2. Model ACE (Model 1 + drop d11)	1	13	−341.168	1485	−315.168	−263.279	1.42645	1	0.23
3. **Model AE (Model 2 + drop c22)**	**2**	**12**	**−339.030**	**1486**	**−315.030**	**−267.133**	**2.13736**	**1**	**0.14**

ctm = compared to model; ep = estimated parameters; −2LL = minus twice the log-likelihood; df = degrees of freedom; AIC = Akaike Information Criterion: −2LL−2df; BIC = Bayesian Information Criterion, −2LL + log((n + 2)/24); diffLL = (−2LL submodel) − (−2LL full model); Δdf = (df submodel) − (df full model); A = additive genetic influence; C = shared environmental influence; D = non-additive genetic influence; E = unique environmental influence; d11 = non-additive genetic factors influencing the first phenotype (e.g., ADHD); c22 = shared environmental factors influencing the second phenotype (e.g., ED). The best-fitting model is printed in bold typeface.

**Table 4:** Standardized genetic and environmental components of variance and covariance of ADHD and ED, and genetic and environmental correlations, deriving from the best-fitting model.

Standardized Components		
	A	E
Vp (ADHD)	0.43 (0.30–0.53)	0.57 (0.47–0.70)
Vp (ED)	0.76 (0.70–0.81)	0.24 (0.19–0.30)
Cov (ADHD-ED)	0.86 (0.75–0.95)	0.14 (0.05–0.25)
Genetic and environmental correlations		
ADHD-ED	r_a_ = 0.92 (0.82–1)	r_e_ = 0.23 (0.19–0.30)

Vp = phenotypic variance; Cov = covariance; A = additive genetic influence; E = unique environmental influence; r_a_ = additive genetic correlation; r_e_ = unique environmental correlation. Numbers in parentheses are 95% confidence intervals. Results are adjusted by age and sex.

**Table 5:** Bivariate model-fitting analysis with sub-model comparisons for CDS and ED.

Model	ctm	ep	−2LL	df	AIC	BIC	diffLL	Δdf	*p*
Model ACDE	-	14	−853.738	1477	−825.738	−769.858	-	-	-
2. Model ACE (Model 1 + drop d11)	1	13	−852.481	1478	−826.481	−774.592	1.25685	1	0.26
3. **Model AE (Model 2 + drop c22)**	**2**	**12**	**−849.167**	**1479**	**−825.1675**	**−777.269**	**3.31440**	**1**	**0.07**

ctm = compared to model; ep = estimated parameters; −2LL = minus twice the log-likelihood; df = degrees of freedom; AIC = Akaike Information Criterion,−2LL−2df; BIC = Bayesian Information Criterion, −2LL + log((n + 2)/24); diffLL = (−2LL submodel) − (−2LL full model); Δdf = (df submodel) − (df full model); A = additive genetic influence; C = shared environmental influence; D = non-additive genetic influence; E = unique environmental influence; d11 = non-additive genetic factors influencing the first phenotype (e.g., ADHD); c22 = shared environmental factors influencing the second phenotype (e.g., ED). The best-fitting model is printed in bold typeface.

**Table 6:** Standardized genetic and environmental components of the variance and covariance of CDS and ED, and genetic and environmental correlations, derived from the best-fitting model.

	Standardized Components	
	A	E
Vp (CDS)	0.29 (0.16–0.41)	0.71 (0.59–0.84)
Vp (ED)	0.77 (0.70–0.81)	0.23 (0.19–0.30)
Cov (CDS-ED)	0.81 (0.66–0.95)	0.19 (0.05–0.34)
	Genetic and environmental correlations	
CDS-ED	r_a_ = 0.81 (0.64–1)	r_e_ = 0.22 (0.06–0.36)

Vp = phenotypic variance; Cov = covariance; A = additive genetic influence; E = unique environmental influence; r_a_ = additive genetic correlation; r_e_ = unique environmental correlation. Numbers in parentheses are 95% confidence intervals. Results are adjusted by age and sex.

## Data Availability

The data that support the findings of this study are available from the corresponding author upon reasonable request.
